# Neutrophil-to-Lymphocyte Ratio and Blood Eosinophil Levels As Inflammatory Indicators in Smoker and Non-smoker Young Adult Patients With Chronic Obstructive Pulmonary Disease at a Tertiary Care Hospital

**DOI:** 10.7759/cureus.56212

**Published:** 2024-03-15

**Authors:** Shah Mohammad Abbas Waseem, Imrana Masood, Anwar H Siddiqui, Mohd Amir, Syed Hilal Hussain, Syed Haider Mehdi Husaini

**Affiliations:** 1 Physiology, Jawaharlal Nehru Medical College, Aligarh Muslim University, Aligarh, IND; 2 Pulmonary Medicine, Jawaharlal Nehru Medical College, Aligarh Muslim University, Aligarh, IND; 3 Medicine, Jawaharlal Nehru Medical College, Aligarh Muslim University, Aligarh, IND

**Keywords:** lymphocytes, neutrophils, copd, non-smokers, blood cell count, smokers, nlr, inflammation, spirometry, biomarkers

## Abstract

Background

Inflammatory markers are elevated in chronic obstructive pulmonary disease (COPD) and can be quantified to detect severity, prognosis, mortality risk, and response to treatment. However, the estimation costs are high. The blood neutrophil-to-lymphocyte ratio (NLR) and eosinophil levels are emerging as biomarkers in COPD, yet there is a paucity of data.

Aim and objectives

This study was designed to elucidate the roles of the NLR and eosinophil levels in smokers and non-smokers with stable COPD male subjects, correlating them with lung functions.

Materials and methods

A prospective observational clinical study was conducted from January to June 2023, after receiving approval from the Institutional Ethics Committee, on 73 COPD patients aged 30-60 years who gave voluntary informed consent. Complete blood counts and spirometry were performed. Patients with a forced expiratory volume in one second (FEV1) % predicted <70% and an FEV1/forced vital capacity (FVC) % <70% based on the pulmonary function test (MIR Spirolab) were included. They were further divided into mild (n=10), moderate (n=27), severe (n=26), and very severe (n=10) categories as per the Global Initiative for Chronic Obstructive Lung Disease (GOLD) guidelines. Subjects were also categorized into smoker (n=45) and non-smoker (n=28) groups. The complete blood count was analyzed using an automated analyzer (Beckman Coulter). Analysis was also carried out with an NLR of more or less than three. A P-value of less than 0.05 was considered significant.

Results

Smokers constituted 61.65% (n=45) of the subjects, and non-smokers 38.35% (n=28). Among smokers, 17.78% had very severe airflow obstruction. In all COPD subjects (n=73), lymphocytes, eosinophils, and lung functions were lower in the group where the NLR was greater than three. NLR in smokers (3.52±1.43) was higher than in non-smokers (3.39±0.94). In non-smokers (n=28), blood eosinophils and lymphocytes were elevated. In smokers (n=45), blood neutrophils, monocytes, and basophils were increased. Smokers showed a non-significant increase in RBC, mean corpuscular volume (MCV), and mean corpuscular hemoglobin (MCH). Neutrophils, monocytes, eosinophils, and NLR increased with disease severity. NLR negatively correlated with FEV1 (r=-0.350, p=0.034) and positively with pack-years (r=0.546, p<0.001) in smokers. NLR negatively correlated with eosinophils, FVC, FEV1/FVC, and FEV1 % predicted. In all COPD subjects (n=73), NLR negatively correlated with blood eosinophils (r=-0.184, p=0.12), BMI, and lung functions.

Conclusion

NLR is elevated in COPD subjects and can serve as a marker of inflammation and a predictor of the risk and severity of airflow limitation. NLR correlates both positively and negatively with pack-years and lung functions, respectively.

## Introduction

Chronic obstructive pulmonary disease (COPD) is a leading cause of mortality and morbidity worldwide, with a pooled prevalence estimated to be 7.4% in India [1]. Literature suggests that the prevalence rate of COPD in Indian males ranges from 2 to 22%, and among males over the age of 35, the rate is 5%. The burden and socio-economic cost of COPD are high [2]. COPD burden appears higher in males, urban areas, and the Northern regions of India [1]. Smoking is recognized as a significant risk factor for COPD [3]; however, not all smokers develop COPD, with studies reporting that 55% of Indians with COPD are non-smokers [4]. Smoking contributes to inflammation, oxidant-antioxidant imbalance, and metabolic disturbances. Inflammation and oxidative stress, implicated in the pathophysiology of COPD, are exacerbated by smoking-induced immunity disruption and severity correlation. Furthermore, inflammation is aggravated by an increase in nitric oxide and an increase in neutrophils and proteinases, resulting in lung function deterioration. Airway inflammation, implicated in COPD pathophysiology, involves CD8+ T lymphocytes, macrophages, and neutrophils. Anti-protease deficiency leads to increased protease activity, causing tissue destruction in COPD. A correlation exists between neutrophils and the severity of airflow limitation. Low histone deacetylase levels in macrophages lead to the upregulation of matrix metalloproteinases and inflammatory markers like IL-8 and TNF-α [5].

The upregulation of inflammatory pathways increases pro-inflammatory cytokines such as TNF-α, IL-6, IL-8, and IL-1β, which play crucial roles in COPD pathophysiology [6]. Therefore, inflammatory markers are elevated in COPD and can be quantified to detect severity, prognosis, mortality risk, and response to treatment, albeit with high cost. Conversely, the neutrophil-lymphocyte ratio (NLR) is a cost-effective method widely used as a marker in COPD. Literature suggests a correlation of NLR with lung functions and its role as an independent marker of prognosis and acute exacerbation in COPD [7]. Also, blood eosinophil levels are associated with severity and may predict the response to steroid treatment, though reports are contradictory and warrant further exploration [8]. A blood eosinophil count greater than two percent may predict exacerbation risk in COPD and is used as a cutoff to diagnose eosinophilia in COPD [9]. High to low eosinophil counts have been reported in COPD patients [10], with counts increasing with the severity of COPD. Blood eosinophils have been included in the guidelines for COPD treatment, generating significant interest in recent years. An NLR greater than three is considered high [8].

In light of the above discussion [7-10], NLR and blood eosinophils have the potential to serve as effective biomarkers in COPD. They are useful as markers and predictors of severity, inflammation, prognosis, and response to treatment. These biomarkers are easily quantifiable and can thus be used to predict clinical outcomes and the severity of the disease. The present study was designed to investigate the NLR and eosinophil levels in smokers and non-smokers with stable COPD male subjects and to correlate these levels with lung functions. Subjects with COPD were classified into grades according to the Global Initiative for Chronic Obstructive Lung Disease (GOLD) [11].

## Materials and methods

This descriptive cross-sectional study was conducted between January and June 2023, recruiting seventy-three male subjects diagnosed with COPD according to the GOLD criteria [11] from the OPD of Pulmonary Medicine. They were further divided into smokers (n=45) and non-smokers (n=28). The study received approval from the Institutional Ethics Committee of Jawaharlal Nehru Medical College and Hospital, Aligarh Muslim University, Aligarh, India (IEC JNMC/710 dated 23/09/2022). Written informed consent was obtained from all study subjects, and the confidentiality of the data was maintained.

Inclusion criteria

Stable COPD male subjects (30-60 years of age) diagnosed as per the GOLD criteria with a history of chronic cough or sputum for at least the past three months of the two consecutive years, patients having forced expiratory volume during first second (FEV1) % predicted <70% and FEV1/FVC % <70% (where FVC is forced vital capacity) based on pulmonary function test (performed using the MIR Spirolab) as per the GOLD, and subjects who were willing to participate and consented were included in the study.

Subjects included in the study were further divided into mild (n=10), moderate (n=27), severe (n=26), and very severe (n=10) categories as per GOLD guidelines. Among the smoker COPD subjects (n=45), the distribution was mild (n=8, 17.78%), moderate (n=14, 31.11%), severe (n=15, 33.33%), and very severe (n=8, 17.78%) as per GOLD [11]. In the non-smoker COPD group (n=28), the presence of mild, moderate, severe, and very severe airflow obstruction was reported in 2 (7.14%), 13 (46.43%), 11 (39.29%), and 2 (7.14%) subjects, respectively.

Exclusion criteria

Subjects under 30 or over 60 years of age, those not giving consent, suffering from lung diseases other than COPD, with a history of congestive heart failure (CHF), chronic kidney disease (CKD), anemia, thyroid disorder, worm infestations, or with a history of substance abuse like alcohol were excluded from the study.

A five-milliliter blood sample was drawn under aseptic conditions, and the complete blood count was analyzed using an automated analyzer (Beckman Coulter). The NLR was calculated as the ratio of neutrophils to lymphocytes, and the analysis also considered NLR values of more or less than three.

Statistical analysis

The data were analyzed using Microsoft Excel and SPSS version 24.0 (IBM Corp., Armonk, NY, USA). Values were expressed as mean ± SD. The number and percentage of COPD subjects were reported for smokers and non-smokers. The data of the study groups were compared using the Chi-square test, ANOVA, and student's unpaired t-tests. Pearson’s correlation was used to find the association of NLR with study variables. P-values of less than 0.05 were considered significant.

## Results

The study was conducted between January and June 2023, during which the data of 73 COPD subjects, diagnosed based on the GOLD criteria, were collected. The study subjects were divided into two groups: smokers (n=45) and non-smokers (n=28), for further analysis.

The mean age (years) and BMI (kg/m²) of the COPD subjects (n=73) were 48.54±5.34 and 21.32±2.4, respectively. The mean value of the Neutrophil-to-Lymphocyte Ratio (NLR) was found to be 3.46±1.23. The mean values of lung functions, namely FEV1 (L), FVC (L), FEV1/FVC (%), and FEV1 % predicted (L/min), were found to be 2.16±0.79, 1.55±0.71, 67.59±19.12, and 57.39±9.76, respectively, with FEV1 % predicted being 3.47±1.43 (Table 1).

**Table 1:** Results of study variables in COPD subjects (n=73). Data was expressed as mean ± SD. NLR was expressed as ratio of neutrophil and lymphocyte counts. FEV1: Forced expiratory volume in one second; FVC: Forced vital capacity; PEFR: Peak expiratory flow rate; NLR: Neutrophil-to-lymphocyte ratio.

Study variables	Mean ± SD (range)
Age (years)	48.54±5.34
BMI (Kg/m2)	21.32±2.4
FEV1 (L)	2.16±0.79
FVC (L)	1.55±0.71
FEV1/FVC %	67.59±19.12
FEV1 % Predicted	57.39±19.76
PEFR (L)	3.47±1.43
NLR	3.46±1.23

The complete blood count was performed on the 73 COPD subjects using an automated analyzer. The differential white blood cell count revealed that the mean values for granulocytes, namely neutrophils (%), eosinophils (%), and basophils (%), were 68.95±6.47, 3.13±1.96, and 0.16±0.33, respectively. The mean values for agranulocytes, namely lymphocytes (%) and monocytes (%), were found to be 24.10±5.13 and 4.90±2.60, respectively. The mean platelet count, expressed in lacs, was 2.01±0.59 (Table 2).

**Table 2:** Blood cell counts in study participants with COPD (n=73). Data is expressed as mean ± SD. COPD: Chronic obstructive pulmonary disease.

Blood cells(%)	Mean ± SD
Neutrophils (%)	68.95 ±6.47
Lymphocytes (%)	24.10±5.13
Eosinophil(%)	3.13±1.96
Monocytes(%)	4.9±2.60
Basophils(%)	0.16±0.33
Platelets (lacs)	2.01±0.59

COPD subjects (n=73) were divided into two groups: 45 (61.65%) were smokers, and 28 (38.35%) were non-smokers, respectively. The severity of airflow obstruction in each group was tested and categorized into grades according to the GOLD criteria. In the smoker group, 8 (17.78%), 14 (31.11%), 15 (33.33%), and 8 (17.78%) subjects had mild to very severe obstruction, respectively. In the non-smoker group, 2 (7.14%), 13 (46.43%), 11 (39.29%), and 2 (7.14%) subjects had mild to very severe COPD, respectively. According to the chi-square test, the difference was insignificant (p=0.07) (Table 3).

**Table 3:** Comparison of smoker and non-smoker COPD groups according to severity grades of airflow obstruction. Categorical variables are expressed as frequency and percentage. Differences are compared using the chi-square test, with the P-value shown in parentheses (*P >0.05). Parentheses ($) show pack years in the smoker COPD group as mean ± SD (15.04 ± 7.94). COPD: Chronic obstructive pulmonary disease.

COPD grade	Smokers (n=45)^$^ Number (%)	Non-smokers(n=28) Number (%)	P-value
Mild (n=10)	8 (17.78)	2 (7.14)	Chi square=6.84 df=3 P=0.07*
Moderate (n=27)	14 (31.11)	13 (46.43)	
Severe (n=26)	15 (33.33)	11 (39.29)	
Very Severe (n=10)	8 (17.78)	2 (7.14)	

Blood cells (neutrophils, eosinophils, and lymphocytes) and pulmonary functions (FEV1, FVC, and FEV1/FVC) were compared based on an NLR greater than and less than three. In groups with an NLR greater than three, the mean value of neutrophils was higher (74.21±4.05) than in those with an NLR less than three (65.29±5.20), and the difference was significant (p<0.001). The difference in blood lymphocytes between the two groups was significant (p<0.001), with mean values lower in the group with an NLR more than three (19.85±3.02) compared to the group with an NLR less than three (27.06±4.11). Similarly, blood eosinophils were significantly lower (3.57±1.74) in the group with an NLR greater than three compared to the group with an NLR less than three (4.46±1.53) (p=0.005). No significant difference was observed in lung functions between the two groups, although FVC, FEV1, and FEV1/FVC % were lower in the COPD group with an NLR greater than three (Table 4).

**Table 4:** Difference in blood cells and pulmonary functions based on NLR value in COPD patients (n=73). Data are presented as mean ± SD. The P-value is shown in parentheses (*P <0.05). NLR was compared using an unpaired Student's t-test based on values greater and less than three. FEV1: Forced expiratory volume in one second; FVC: Forced vital capacity; NLR: Neutrophil-to-lymphocyte ratio; COPD: Chronic obstructive pulmonary disease.

Variables	NLR >3	NLR <3	P-value
Neutrophil (%)	74.21±4.05	65.29±5.20	<0.001*
Lymphocyte (%)	19.85±3.02	27.06±4.11	<0.001*
Eosinophil (%)	3.57±1.74	4.46±1.53	0.005*
FVC (L)	1.43±0.65	1.63±0.75	0.242
FEV1 (L)	1.96±0.72	2.29±0.80	0.07
FEV1/FVC (%)	66.94±20.71	69.04±18.16	0.811

Table 5 shows the differences in the mean values of age, BMI, lung functions (FEV1, FVC, FEV1/FVC %, and FEV1 % predicted), and NLR between the smoker (n=45) and non-smoker (n=28) groups. No significant difference was found in mean age (49.37±6.86 vs. 47.08±9.17, p=0.346) or BMI (24.33±2.02 vs. 25.12±1.63, p=0.235) between the two groups. The mean values of NLR were significantly higher in smokers (3.52±1.43) compared to non-smokers (3.39±0.94) (p=0.032). Significant differences were observed in FEV1 (1.96±0.81 vs. 2.04±0.68, p=0.017), FVC (1.30±0.63 vs. 1.86±0.70, p=0.001), FEV1/FVC % (66.25±17.31 vs. 69.16±14.35, p=0.027), and FEV1 % predicted (51.36±20.37 vs. 65.12±16.15, p=0.003), with values lower in smokers compared to non-smokers (Table 5).

**Table 5:** Comparison of pulmonary functions and NLR between smoker and non-smoker COPD group. Data are presented as mean ± SD. The P-value is shown in parentheses (*P <0.05). Variables were compared using an unpaired Student's t-test between the smoker (n=45) and non-smoker (n=28) COPD groups. FEV1: Forced expiratory volume in one second; FVC: Forced vital capacity; NLR: Neutrophil-to-lymphocyte ratio; COPD: Chronic obstructive pulmonary disease.

Variables	Smoker COPD (n=45)	Non Smoker COPD (n=28)	P-value
Age (years)	49.37±6.86	47.08±9.17	0.346
BMI (Kg/m2)	24.33±2.02	25.12±1.63	0.235
FVC (Liters)	1.30±0.63	1.86±0.70	0.001*
FEV1 (Liters)	1.96±0.81	2.04±0.68	0.017*
FEV1/FVC (%)	66.25±17.31	69.16±14.35	0.027*
FEV1 % predicted	51.36±20.37	65.12±16.15	0.003*
NLR	3.52±1.43	3.39±0.94	0.032*

Table 6 shows that blood cells, namely eosinophils (3.82±1.50 vs. 4.07±2.463, p=0.596) and lymphocytes (23.11±4.92 vs. 24.87±5.22, p=0.149), were non-significantly higher in non-smokers compared to the smoker COPD group. In the smoker group, a non-significant increase in neutrophils (69.81±6.10 vs. 67.85±6.85, p=0.201) and monocytes (5.36±2.72 vs. 4.31±2.34, p=0.087) was observed in comparison to non-smokers. Basophils were significantly higher in smokers (2.40±0.41) compared to non-smokers (1.01±0.35, p=0.028). Blood indices such as mean corpuscular volume (MCV; 89.56±8.56 vs. 83.45±6.54, p=0.876), mean corpuscular hemoglobin (MCH; 29.65±3.45 vs. 28.43±2.10, p=0.239), and RBC (5.32±0.02 vs. 4.88±0.21, p=0.365) were non-significantly higher in the smoker COPD group compared to the non-smoker COPD group, whereas mean corpuscular hemoglobin concentration (MCHC) was non-significantly lower in smokers (321.45±22.23 vs. 331.32±17.86, p=0.112) in comparison to non-smokers (Table 6).

**Table 6:** Comparison of blood cells and indices between smoker and non-smoker COPD groups. Data are presented as mean ± SD. The P-value shown in parentheses (*P <0.05) signifies a significant difference between blood basophils in the smoker and non-smoker groups. MCV: Mean corpuscular volume; MCH: Mean corpuscular hemoglobin; MCHC: Mean corpuscular hemoglobin concentration; COPD: Chronic obstructive pulmonary disease.

Blood parameters	Smoker COPD (n=45) Mean ±SD	Non-smoker COPD (n=28) Mean ±SD	P-value
Neutrophils (%)	69.81±6.10	67.85±6.85	0.201
Lymphocytes (%)	23.11±4.92	24.87±5.22	0.149
Eosinophils (%)	3.82±1.50	4.07±2.463	0.596
Monocytes (%)	5.36±2.72	4.31±2.34	0.087
Basophils (%)	2.40±0.41	1.01.±0.35	0.028*
Platelets (lacs)	2.04±0.63	1.96±0.54	0.571
MCV (fL)	89.56±8.56	83.45±6.54	0.876
MCH (pgm)	29.65±3.45	28.43±2.10	0.239
MCHC (g/L)	321.45±22.23	331.32±17.86	0.112
RBC (millions/mm^3^)	5.32±0.02	4.88±0.21	0.365

Among the COPD subjects (n=73) divided as per the GOLD grades, a non-significant increase in blood neutrophils, monocytes, and eosinophils from mild to very severe airflow obstruction was observed. However, among the groups, NLR was found to rise with the severity of obstruction (mild=2.67±0.99, moderate=3.32±0.89, severe=3.44±1.11, and very severe=4.71±1.76), and the difference was significant (p=0.001). Similarly, blood basophils were higher in very severe obstruction (0.52±0.69) compared to mild (0.08±0.17), moderate (0.11±0.22), and severe obstruction (0.18±0.20), with the difference being significant (p=0.002) (Table 7).

**Table 7:** Differences in blood cells and NLR as per the grade of COPD (n=73). Data are presented as mean ± SD. The P-value is shown in parentheses (*P <0.05). Variables were compared using ANOVA within the COPD groups based on the severity of airflow obstruction. COPD: Chronic obstructive pulmonary disease; NLR: Neutrophil-to-lymphocyte ratio.

Variable	COPD Grade (GOLD)				P-value
	Mild(n=10)	Moderate (n=27)	Severe(n=26)	Very Severe(n=10)	
Neutrophils (%)	66.50±6.77	68.46±6.99	69.74±5.76	70.70±6.56	0.447
Lymphocytes (%)	25.80±6.42	24.02±5.81	23.89±4.70	23.1±2.74	0.699
Monocyte (%)	4.36±2.40	4.63±2.58	5.83±2.19	6.1±2.81	0.189
Eosinophils (%)	3.41±1.73	3.59±1.86	4.32±2.31	4.27±2.01	0.424
Basophils (%)	0.08±0.17	0.11±0.22	0.18±0.20	0.52±0.69	0.002*
NLR	2.67±0.99	3.32±0.89	3.44±1.11	4.71±1.76	0.001*

In the smoker COPD group, NLR was higher in very severe obstruction (4.98±1.8) compared to mild (2.78±1.01), moderate (3.29±0.92), and severe (3.66±1.24) grades, and the difference was found to be significant (p=0.011). A significant difference (p<0.001) was also observed in pack years based on the severity of obstruction, with pack years being higher in subjects with very severe obstruction (26.13±2.21). The difference in blood eosinophils among the group was non-significant (p=0.267), with higher eosinophils in mild COPD (4.21±0.58) compared to moderate (3.75±1.34), severe (3.53±1.30), and very severe (2.88±1.84) grades of obstruction (Table 8).

**Table 8:** Difference in pack years, NLR, and eosinophil counts among smoker COPD patients (n=45) according to the grade of airflow obstruction. Data are presented as mean ± SD. P-values are shown in parentheses. *P < 0.05. Variables were compared using ANOVA within the smokers' COPD group based on the severity of airflow obstruction. NLR: Neutrophil-to-lymphocyte ratio; COPD: Chronic obstructive pulmonary disease.

Variable	Mild (n=08)	Moderate (n=14)	Severe (n=15)	Very severe (n=08)	P-value
Pack years	6.26±3.96	11.68±3.50	16.90±5.20	26.13±2.21	<0.001*
NLR	2.78±1.01	3.29±0.92	3.66±1.24	4.98±1.8	0.011*
Eosinophils (%)	4.21 ±0.58	3.75±1.34	3.53±1.30	2.88±1.84	0.267

In the smoker COPD group, there was a significant negative correlation of NLR with FEV1 (r=-0.350, p=0.034) and a positive correlation with pack years (r=0.546, p<0.001). Additionally, non-significant negative correlations of NLR with FVC (r=-0.213, p=0.206), FEV1/FVC % (r=-0.081, p=0.633), FEV1% predicted (r=-0.120, p=0.48), and eosinophils (r=-0.049, p=0.772) were observed (Table 9).

**Table 9:** Correlation of NLR with study parameters in smoker COPD patients (n=45). P-values are shown in parentheses. *P < 0.05 indicates a significant negative Pearson correlation between FEV1 and NLR in the smoker COPD group. #P < 0.05 indicates a significant positive Pearson correlation between pack-years and NLR in the smoker COPD group. FEV1: Forced expiratory volume in one second; FVC: Forced vital capacity; NLR: Neutrophil-to-lymphocyte ratio; COPD: Chronic obstructive pulmonary disease.

Variable	R-value	P-value
FVC (liters)	-0.213	0.206
FEV1 (liters)	-0.35	0.034*
FEV1/FVC (%)	-0.081	0.633
FEV1 percentage predicted	-0.12	0.48
Pack years	0.546	<0.001#
Eosinophils (%)	-0.049	0.772

In all COPD subjects (n=73), the correlation of NLR with blood eosinophils was non-significantly negative (r=-0.184, p=0.12). A non-significant negative correlation of NLR with BMI (r=-0.050, p=0.673) and FEV1 % predicted (r=-0.195, p=0.098) was observed, whereas the correlation of NLR with FEV1 (r=-0.378, p=0.001) and FVC (r=-0.298, p=0.011) was significantly negative (Table 10).

**Table 10:** Correlation of the NLR with study parameters in COPD patients (n=73). P value shown in parentheses: *P < 0.05 indicates a significant negative Pearson correlation between FVC, FEV1, and NLR in COPD subjects (n=73). FEV1: Forced expiratory volume in one second; FVC: Forced vital capacity; NLR: Neutrophil-to-lymphocyte ratio; COPD: Chronic obstructive pulmonary disease.

Variable	R-value	P-value
FVC (liters)	-0.298	0.011^*^
FEV1 (liters)	-0.378	0.001*
BMI (Kg/m2)	-0.050	0.673
FEV1/FVC (%)	-0.019	0.875
FEV1 percentage predicted	-0.195	0.098
Eosinophils (%)	-0.184	0.12

## Discussion

In our study, 45 (61.65%) subjects were found to be smokers and 28 (38.35%) non-smokers, respectively, aligning with the observed trend. Literature suggests that COPD prevalence in non-smokers varies between 22 and 51% [12]. However, it may range from 25-45%, and in India, according to available data, between 9.4-68.6%. Biomass fuel exposure is a significant risk factor for COPD, and in India, due to its use, the prevalence of COPD among non-smokers is higher (50%) than in Western Nations [13]. The present study can’t entirely rule out biomass fuel exposure. Also, the higher prevalence of smoking in males may be a factor for a higher percentage of smokers with COPD in our study, as all the subjects were males. The mean age of the subjects was 48.54±5.34 years. 61.65% of the study subjects were smokers, with mean pack-years of 15.04±7.94. Evidence suggests that advancing age, smoking, and pack-years are risks for COPD [14]. In our study, in the smoker COPD group (n=45), 29 (64.45%) had moderate (n=14, 31.11%) to severe (n=15, 33.33%) COPD as per GOLD grade. In the non-smoker group (n=28), moderate (n=13, 46.43%) to severe (n=11, 39.29%) grades were found in 24 (85.71%) subjects, respectively. Lung functions were lower in smokers compared to non-smokers, in line with an earlier study. The results align with a previous study in which moderate grades were higher in non-smokers than in smokers [15]. However, in contradiction to the previous survey, there were more severe grade subjects in non-smokers than in smokers. These results may be attributed to the low sample size and the fact that exposure to biomass fuel and secondhand smoke cannot be entirely ruled out. It was not considered in the present design, thus a limitation and confounding factor. Apart from that, other risk factors for COPD [16] must be thoroughly ruled out in future studies to obtain better results.

In our study, the mean value of NLR was significantly higher (p<0.05) in the smoker COPD group (3.52±1.43) compared to non-smokers (3.39±0.94) with COPD. Pulmonary functions were significantly lower in the former group. Neutrophils and monocytes were non-significantly higher, and lymphocytes and eosinophils non-significantly lower in smokers compared to non-smokers, whereas blood basophils in COPD smokers were significantly higher (p=0.028) compared to non-smokers. The results could be explained by the literature that suggests inflammation leads to the recruitment of neutrophils and eosinophils, which in turn, upon activation, result in the release of matrix metalloproteinase, myeloperoxidases, elastase, proteinases, and cathepsin G, all implicated in airway inflammation, tissue destruction, and thus playing a role in the pathophysiology of COPD. In recent years, NLR has been utilized as a diagnostic and prognostic marker in COPD, as well as a predictor of acute exacerbation. Research suggests that patients with low NLR are at less risk of exacerbation and mortality [17].

In our study, with increased COPD severity in all study subjects, i.e., 73, NLR, blood neutrophils, eosinophils, basophils, and monocytes were increased, whereas lymphocyte counts were reduced. Research suggests that smoking is associated with low-grade inflammation, which accounts for the increase in WBC counts [18]. Reduced lymphocyte levels in COPD indicate a stress response, and a decline in lymphocyte count is associated with lung function decline. With increased COPD severity, there is an increase in neutrophils and macrophages with a resultant decrease in lymphocyte counts. Cortisol, inflammation, and catecholamines result in a decline in lymphocyte counts [19]. However, studies have shown elevated levels of monocytes and lymphocytes in smokers with COPD compared to non-smokers [20]. Smoking stimulates the granulocyte-monocyte colony-stimulating factor (GM-CSF), resulting in monocytosis due to enhanced myelopoiesis. The smoking-induced increase in monocytes is associated with a decline in lung functions and is linked with COPD. Monocytes thus can be used as a marker in COPD to predict prognosis [21].

In an earlier study, basophil, monocyte, and neutrophil counts were elevated in COPD smokers. Increases in leukocytes and lymphocytes have also been reported. Platelets are increased with smoking, as reported in earlier studies. With smoking cessation, the values are known to decline toward normal. Inflammation and oxidative stress are central to the pathophysiology of COPD, leading to an increase in NLR, which is associated with a decline in lung function. However, there are contradictory findings, with studies suggesting that NLR predicted the difference between moderate and severe COPD [22].

Previous research suggests a negative correlation between NLR and FEV1. However, contradictory reports are available regarding the correlation of NLR with COPD severity. Both positive linear relations, as well as no relation between NLR and stages of COPD as per the GOLD, are reported. Results from previous studies suggest that in both stable and acute stages of COPD, NLR was high in patients with low levels of eosinophil. Patients with low eosinophils are reported to respond poorly to corticosteroid treatment, and in stable COPD patients, eosinophils correlate negatively with bacterial infections. Also, a high NLR is an independent marker for mortality in COPD patients. Evidence suggests a negative correlation between eosinophils and FEV1 levels in COPD patients [5, 7].

Results of our study show that in the smoker COPD group, eosinophils were found to decline with the rise in COPD severity. Evidence from previous studies suggests that low eosinophil counts are associated with a longer stay in the hospital, exacerbation, a higher risk of infections, and poor prognosis over a one-year follow-up compared to those with higher eosinophil counts [23]. As per the aforementioned study, NLR correlates with the severity of airway obstruction and eosinophils, declining with increasing COPD severity. This could explain the observation in our research that in COPD subjects with NLR greater than three, there was a significant increase and decrease in neutrophils and eosinophils, respectively, compared to the group with NLR less than three. Reports suggest that an NLR of more than 2.94 is an independent predictor of future acute exacerbation during a year-long follow-up study [8]. Another study indicated that an NLR greater than 2.8 is associated with a high risk of hospitalization in patients with COPD. An increase in NLR is related to an increase in neutrophils and is considered a marker for the use of antibiotics in COPD [24].

In our study, MCV, MCH, RBC, and platelet counts were non-significantly higher in smokers compared with non-smoker COPD subjects. MCV and RBC are reportedly increased in smokers. The increase in MCV is attributed to the smoking resultant oxidant-antioxidant imbalance, which causes damage to the membrane, and also to acetaldehyde produced in the smoke, causing toxic damage to bone marrow. Low to no change in MCV and MCH are reported in other studies [25]. Smoking results in a compensatory increase in hemoglobin due to carboxyhemoglobin formation, which reduces the oxygen-carrying capacity. In smokers, hemoglobin, MCV, and MCH are found to be raised. Carboxyhemoglobin results in the compensatory increase in the secretion of the hormone erythropoietin from the kidney, increasing erythropoiesis. Also, the presence of nicotine in tobacco stimulates the release of the hormone cortisol and catecholamines, which, along with inflammation, results in leukocytosis. Inflammation also increases the permeability of the capillaries and upregulates the adhesion molecules, resulting in increased adhesion of the platelets and leukocytes. Smoking, along with inflammation, thus increases the number of leukocytes in circulation [26]. Smoking-induced oxidative stress increases thromboxane A2 (TXA2), thrombopoietin, and isoprostanes, which increase platelet counts. Low-to-high platelet counts are reported in smokers. The duration and severity of smoking affect the platelet count. In our study of smokers and overall COPD subjects, NLR showed a negative correlation with lung functions and eosinophil levels, whereas, in the smoker group, a significant positive correlation (r=0.546, p<0.001) of NLR was found with pack years. A negative correlation between NLR and eosinophils was reported in an earlier study. The levels of eosinophils are reported to decline in smokers, possibly due to the retention or recruitment of eosinophils in the lung tissue [27].

The present study found a negative correlation between NLR and BMI, which could be attributed to inflammation, as reported in an earlier study [28]. In our research, NLR in smokers was raised compared to non-smokers, and the BMI was lower in the former group. A significant negative correlation of NLR with BMI and FEV1 has been reported in the earlier study. NLR increases with airflow severity, indicating the role of neutrophils in inflammation and oxidative damage-induced emphysema changes in the lung [29]. Studies have reported a negative correlation between NLR and BMI. COPD is associated with inflammation and muscle atrophy due to disruption in the regenerative capacity of satellite cells [30].

Limitations and future suggestions

The cross-sectional design limits the establishment of causal relations. The relatively smaller sample size also limits drawing definitive conclusions, especially when stratifying by severity and smoking status. Additionally, the inclusion of males only is a limitation regarding the generalization of findings and represents a selection bias. Thus, a prospective follow-up study in COPD subjects with a larger sample size (including both males and females, and conducting comparative analysis between the two genders) will yield utility of NLR and eosinophils as an inflammatory marker with prognostic and predictive value in lung function decline and COPD exacerbation. Correlation with clinical outcomes and hospital stay duration will yield better results. Also, in non-smokers with COPD, exposure to environmental smoke and genetic factors like anti-protease deficiency and asthma are possible factors related to COPD and need to be explored. The prospective study needs to be designed to study the response to corticosteroids in patients with low to high eosinophil counts and the risk of acute exacerbation, along with possible correlation with eosinophils.

## Conclusions

NLR is raised in COPD subjects and can be used as a marker of inflammation and as a predictor of the risk and severity of airflow limitation. NLR correlates positively and negatively with pack years and lung functions. Tobacco is associated with inflammation and up-regulation of inflammatory cytokines. The cost of estimation is often high, adding to the economic burden of COPD, and thus, effective markers of inflammation need to be explored and researched.

Clinical implications

The study results have possible clinical implications. Blood cell estimation and ratios like NLR can predict the severity of the disease and, if researched prospectively, even the duration of stay, prognosis, and clinical outcomes in COPD. Thus, a follow-up longitudinal study is needed wherein NLR eosinophils are estimated serially and correlated with severity, exacerbation, duration of stay, and prognosis.
